# Defining Adjustment to Address the Missing Link between Refugees and Their Resettlement Communities

**DOI:** 10.3390/ijerph18189902

**Published:** 2021-09-20

**Authors:** Camilla Modesti, Alessandra Talamo

**Affiliations:** Department of Social and Developmental Psychology, Sapienza University of Rome, 00185 Rome, Italy; alessandra.talamo@uniroma1.it

**Keywords:** refugees, adjustment, community development

## Abstract

Background: data from the United Nations High Commissioner for Refugees (UNHCR) show that, in 2020, among 82.4 million refugees, only 251,000 returned to their home countries, indicating the desire for refugees to stay, for the long-term, in their new homelands. The paper contributes to the scientific–psychological debate on the social representation of refugee populations, by studying this population, not simply as “foreigners”, traumatized and resourceless people, but rather focusing on the factors that lead to their positive adjustments within local communities. Method: a scoping review was carried out to explore the phenomenon of adjustment (RQ1) and to identify the factors that foster adjustment among refugees and their resettlement communities (RQ2). A research protocol and eligibility criteria were defined prior to conducting the literature research through the Scopus database. Afterwards, data charting and items were conducted to organize the results. Results: a process of data mapping outlined three dimensions of adjustment—psychological, social, and scholastic. In addition, six macro factors emerged that ease refugee adjustments—context characteristics, time, social integration markers, acculturation, social support, and psychological capital. Results show that adjustment is the result of the inter-relations among sociological and psychological factors. Conclusions: the lack of studies addressing the inner resources of refugees and community participation confirms that research in this field needs a change of paradigm, to identify the resources that refugees use to adjust to their new communities and promote their development.

## 1. Introduction

### 1.1. Questioning the Association between Refugees and Trauma: The Need for a Positive Psychology Approach

The 1951 Refugee Convention defines a refugee as a person “who is unable or unwilling to return to their country of origin owing to a well-founded fear of being persecuted for reasons of race, religion, nationality, membership of a particular social group, or political opinion” [1] (p. 3). One year prior to the definition provided by the Convention, the United Nations High Commissioner for Refugees was founded, to assist Europeans who left their homes because of World War II. For an in-depth historical analysis of refugee reception in Europe, refer to Gatrell’s essay [2].

The UNHCR, as the global agency that deals with the rescuing and resettlement of refugees, has adopted three long-term solutions for the settlement of refugees—voluntary repatriation, local integration, and resettlement [3]. Voluntary repatriation allows refugees to return to their home countries. According to the UNHCR, in 2020, only 251,000 of 82.4 million refugees benefited from this solution [4]. Local integration is another durable solution and consists of progressive inclusion within the asylum country. Even if it is not guaranteed, local integration might conclude with citizenship. According to the UNHCR, over the past decade, 1.1 million refugees were granted citizenship from their asylum countries [5]. Lastly, resettlement consists of relocating refugees from an asylum country to another country that has agreed to permanently admit them. With the resettlement strategy, refugees are granted permanent stay as soon as they arrive in the receiving country. Differently, with the local integration strategy, refugees must apply for asylum and, once acquiring refugee status, refugees must go through a process of social, cultural, economic inclusion. The difference between local integration and resettlement involves the efforts by the host country to include refugees within their local communities. Nevertheless, there are cases, especially for those countries placed at the borders of high-income regions, such as Europe and North America, whereby both resettlement and local integration strategies are adopted.

We reviewed data on the voluntary repatriation of refugees living in asylum countries and resettlement countries; despite the durable solutions adopted by the receiving countries, the host countries often become refugee “resettlement countries” [6]. In addition, both resettlement and local integration bring into question the need to include refugees within local communities, an issue that is impacted not just by bureaucratic solutions, but also by political and economic contexts and public opinion.

In recent years, the reception and social integration of refugees has become central in political debates in western countries, especially after the so-called “refugees’ crisis”. As De Lemos and colleagues suggest [7], such a political debate is mediated by mass media, who play a fundamental role in the social representation of refugees. Studies in this field show that, while right wing media focus their narratives on crime and legality, left wing parties focus on refugee victimization [8]. Nowadays, the term “refugee” has assumed an emotional meaning in addition to the original institutional meaning that had the specific function of ensuring secure shelter for those displaced because of persecution, conflict, violence, and human rights violations. According to the narratives shared by the mass media in western countries, refugees are either people to fear or to rescue [8]. Although qualitatively different, these representations foster an image of refugees as resourceless foreigners who merely consume the host community’s resources produced by natives. (Since studies in the field have underlined the role of words and, more generally, communication play, in fostering public opinion toward immigration [7,8], the authors made the linguistic choice to address members of the resettlement communities as *natives* or *autochthonous* in order to specify that the main difference between them and refugees is the birth-country.) This image hinders the recognition of “refugee belongingness” to the resettlement community and the chance to identify how they actively participate in its development. As Phillips and Pittman [9] note, community development can be considered both a process and an outcome. The authors suggest the following as a comprehensive definition of community development that takes into consideration both aspects of the concept. Community development is “A process: developing and enhancing the ability to act collectively, and an outcome: (1) taking collective action and (2) the result of that action for improvement in a community in any or all realms: physical, environmental, cultural, social, political, economic, etc.” (p. 6). 

Therefore, the objective of this scoping review is to strengthen the scientific–psychological perspective that goes beyond the representation of refugee populations as traumatized and resourceless people, and focuses more on the contributions that such an eclectic group provides to local communities. The paper will also provide scholars, from different disciplines, with insight into the factors that positively impact the social–psychological dynamics that tie refugees with natives in the resettlement countries. 

Refugees are “normal individuals with strengths and resources who have been caught in abnormal situations” [10] (p. 314). Cobb and colleagues [11] make a case for the application of a positive psychology framework to the studies on migrants.

Seligman and Csikszentmihalyi [12], with positive psychology, introduced a new theoretical approach, aimed at exploring the development of human inner strengths. The authors felt that the development of psychology after WWII, with a focus on pathology and diagnosis, and treatment of mental illness, led to a characterization of human beings as passive spectators of external events. To combat this notion, positive psychology involves the study of factors that help individuals achieve optimal psychological health. Positive psychology seeks to both understand and promote well-being, describing the psychological characteristics that promote positive, adaptive behavior, and assessing interventions aimed at enhancing mental wellness [13].

Within the migration field, to complement the significant body of research that investigates the negative effects of adversities faced before, during, and after resettlement through a pathological model, efforts must be made to study the internal and external resources that sustain newcomer adaptations in the host country. A pathological approach to the psychological study of refugees perpetuates the label of refugees as victims—helpless, powerless, and in need of aid. It “denies refugees the role of being social and political actors and agents of their own recuperation” [10] (p. 316). Positive adaptation among immigrants considers, instead, the extent to which they succeed within their groups and within the larger society, in light of the demands placed on them by living in a new culture [14]. Initial research attributes such success to factors, such as “conditions of pre- and post-migration societies, key acculturation processes, community contexts, family contexts, cultural values, and character strengths” [11].

The main objective of positive psychology (regarding refugees) is to understand the characteristics of the many refugees who are able to withstand objectively difficult life experiences without developing mental illness. Such a study includes both the cognitive strategies employed in the face of intense and often long-lasting stressors and the internal psychological factors that protect refugees from developing functional impairment. As such, a positive psychology of refugees involves the study of coping mechanisms, factors promoting resilient responses, and growth or positive personal development driving from adverse experiences. Such an approach does not deny the fact that many refugees suffer severe psychological distress and trauma, but seeks to build from that base to better understand the mechanisms by which the majority of refugees continue to function adequately and achieve satisfactory levels of well-being [10]. 

### 1.2. Adjustment: Linking Refugee Integration with Community Development

Refugees who leave their homelands for security reasons enter their new communities to build a new life. Psychological studies that have addressed refugee issues are divided among those that explore their mental health conditions before, during, and after their resettlement and those that investigate their entrance and inclusion in the resettlement community. According to the World Health Organization [15], community engagement and mental health are strongly inter-related to the point that social exclusion is considered one of the risk factors for poor mental health conditions. 

Including/accepting newcomers into a community could improve their mental health status and foster community cohesion, defined as: “intertwined features of society, which may be described as: (1) the absence of latent social conflict—whether in the form of income/wealth inequality; racial/ethnic tensions; disparities in political participation; or other forms of polarization; and (2) the presence of strong social bonds—measured by levels of trust and norms of reciprocity (i.e., social capital); the abundance of associations that bridge social divisions (”civil society”); the presence of institutions of conflict management (e.g., a responsive democracy, an independent judiciary, and so forth)” [16] (p. 175).

Social cohesion is, in turn, the basis for the creation of collective social capital, defined by sociologist Robert Putnam as “features of social organization, such as networks, norms, and trust, that facilitate coordination and cooperation for mutual benefit” [17] (p. 2). Collective social capital consists of networks of civic engagement that promote mutuality*, cooperation,* and *coordination* (p. 4), as well as a shared awareness about reciprocal trustworthiness. Thus, newcomer inclusion in the resettlement community does not represent a “scope” for its own sake, rather, from a larger perspective, it is functional to the community development. 

Newcomers’ inclusion within the resettlement community has been studied in terms of integration. In psychological studies, the term integration refers to a newcomer’s position within the resettlement community, whereby “integration” indicates that they have reached the prerequisites that make them effective members of the community. This first branch of study addresses integration from a normative point of view. Another branch of psychological studies links the term “integration” to cultural belongingness and considers “integrated” newcomers who successfully navigate among their multiple cultural belongings. This second branch of studies explores the psychological changes that are a consequence of an intercultural contact. We propose that these two dimensions of integration are strongly inter-related.

The first branch of studies is represented by the Ager and Strang model [18]. According to this model, newcomers granted a regular residence permit become integrated once they achieve the following goals: durable employment, stable housing conditions, and the ability to independently navigate among the healthcare and educational systems. The achievement of these four goals is eased by social connections, with the heritage and the resettlement community, and with local institutions, and by the acknowledgement of the local language and culture. According to the authors, a sense of safety and stability within the local community also facilitates the achievement of these four goals. 

The Ager and Strang model captures an important aspect of the resettlement process: achievement of the requisites that allow newcomers to be self-sufficient within the resettlement country. Self-sufficiency is here considered a status of independency from the welfare services, economic self-sufficiency, and connection with the resettlement and heritage communities. Therefore, this model refers to integration as a final stage on the path that brings newcomers to socially adapt to the resettlement community, and to acquire its cultural values. Nevertheless, consistent with Mantovani [19], integration is here considered a process of social and cultural homologation to the receptive community. The newcomer’s background, with its cultural specificities, is not taken into consideration, leaving out a fundamental aspect of the resettlement experience: the psychological experience of crossing different cultures (e.g., national, local, religious, etc.). Migrants, by leaving their origin countries, find themselves in places with different cultural references. One of the issues of the migratory experience is the need to navigate across these different cultures in a way that is functional to the person’s social and psychological adaptation.

Berry’s acculturation model addresses this very topic by exploring the acculturation process, or rather the psychological changes that occur after an intercultural contact. According to Berry, acculturation constitutes two processes: “cultural maintenance (to what extent are cultural identity and characteristics considered to be important, and their maintenance strived for); and contact and participation (to what extent should they become involved in other cultural groups, or remain primarily among themselves)” [20] (p. 9). This definition clarifies that acculturation concerns individuals as much as communities; moreover, it encompasses not only minority immigrant populations, but also the native majority populations.

According to Berry’s model, newcomers become integrated whether they engage with members belonging to the same culture of origin or those belonging to other cultures. Integration is an acculturation strategy that is facilitated by multicultural societies, or rather societies that sustain mutual accommodation between the dominant and minority cultural groups of a community. Indeed, multiculturalism implies an agreement on basic coexistence values and, for the receptive country, the adoption of culture-sensitive measures in national institutions [21]. 

Berry’s merit is to identify a dialectic between the resettlement country’s acculturation culture and the newcomer’s acculturation strategies. This model shows that, in order to integrate, not only the newcomers’ efforts are needed, but also a context of openness toward the reception and inclusion of those who are not natives. Such openness is actualized through inclusion policies that ease intercultural contact, newcomers’ economic and social inclusions, and the chance to actively contribute to the community development. 

The models by Ager and Strang, as well as Berry, explore two different (yet inter-related) dimensions of integration. There is no social integration without the experience of a sense of belongingness toward the resettlement country: a person can be self-sufficient in a place whereby he or she does not experience a sense of belonging. Within this condition, this person’s engagement in the local community may be motivated only by the need to maintain sustainable life conditions. Nevertheless, a newcomer’s subjective belongingness on its own does not fully explain the experience of integration, which is also comprised of a series of requisites to achieve in order to be institutionally recognized as part of the community. 

This consideration confirms that the normative social integration path and the psychological path are inter-related, and that they cannot be fully understood if not integrated together.

Furthermore, these two models implicitly represent integration as a process for its own sake, whereby the achievement of a socially integrated position is the final stage of an inclusion path. In other words, a newcomer’s engagement in a local community is strictly associated to economic and cultural inclusion. From a community perspective, newcomer inclusion and integration is instead aimed at fostering active participation toward the community’s economic, social, and cultural development. While integration is focused on the newcomer’s resettlement path, we propose to use the term “adjustment” in order to study the dialectic process in newcomer inclusion and the development of local communities. 

Adjustment is here intended as a complex process of mutual accommodation among newcomers and the resettlement communities, in that easing the newcomer’s active participation in the resettlement community promotes its development.

Therefore, the term “adjustment”, different from “social integration”, does not imply a defined scope to be reached; rather, it refers to a process that may be continuous over time. This does not imply that new citizens will never become part of their resettlement communities. On the contrary, it suggests that their involvement in local communities is constantly changing and varies according to the contextual characteristics and the progressive participation of new citizens in the community. To clarify, new citizens are here defined as former newcomers who feel a belongingness to their resettlement communities, and are (formally or informally) recognized as effective members.

To verify the appropriateness of this conceptualization of adjustment, a scoping review was carried out with the specific objectives to:

1. Explore the state-of-the art literature on this argument;
2. Align our theoretical proposal with the existing literature on the subject;
3. Advance the development lines for the theoretical and empirical exploration of adjustment.

Therefore, the following two research questions (RQ) were formulated:

1. How is adjustment conceptualized in the literature?
2. What are the factors that foster adjustment among adult refugees and their resettlement communities?

## 2. Materials and Methods

The protocol we adopted to conduct the scoping review followed the PRISMA guidelines [22]. A scoping review is an emergent methodology for literature that:

“… examine the extent (that is, size), range (variety), and nature (characteristics) of the evidence on a topic or question; determine the value of undertaking a systematic review; summarize findings from a body of knowledge that is heterogeneous in methods or discipline; or identify gaps in the literature to aid the planning and commissioning of future research”.

[22] (p. 1)

To address these aims, the scoping review analyzes a wide range of literature in order to identify a select number of papers that specifically address the research objective. Given the breadth of its purpose, a scoping review may take into consideration a variety of research items [23]. After identification of the review rationale and objectives, the scoping review method proceeds by defining a research protocol and eligibility criteria. These should indicate the databases selected to carry out the review (the guidelines suggest using at least one database), including any limits used, the language, and all criteria adopted for selecting the sources of evidence. Once the research is carried out, a process of data charting and items are required to extract, organize, and list the results from the research protocol. 

From February to March 2021, the authors carried out a literature review within the Scopus database. Given the research questions, the keywords "refugees" and "adjustment" were inserted.

The research group deliberately did not search for papers addressing “social integration”, as literature in the field is already systematized in models that provide exhaustive theorizations on the subject. Furthermore, as aforementioned, social integration is merely focused on newcomer homologation to the resettlement community, while the research group was interested in exploring the relationship between the newcomer resettlement path and community development.

In addition, the term resilience, which the American Psychological Association defines as “the process of adapting well in the face of adversity, trauma, tragedy, threats or even significant sources of stress” [24] (par.4), was not inserted for two reasons. First, it recalls potentially traumatic experiences; thus, confirming the pathological model commonly adopted when addressing psychological studies in refugee populations. Second, it is a concept that merely refers to a refugee’s ability, without considering the contextual characteristics and how they impact the positive adaptation of refugees.

Lastly, consistent with our definition of adjustment as a process that is continuative over time, we did not insert the term “adaptation” among the keywords. As the literature highlights, adjustment refers to a process that could be monitored, while adaptation refers to an outcome that could be evaluated [25].

Peer-review papers written in English were included with no time limits for publication. Quantitative, qualitative, and mixed-method studies were included in order to consider different aspects of refugee adjustments. Review and theoretical papers were also included. Table 1 shows the inclusion and exclusion criteria of the search protocol—a preliminary research identified 216 papers.

In order to answer the first research question, eligible papers had to specifically address and define the concept of adjustment in refugee populations. At this stage of the literature analysis, refugee age did not represent an eligibility criterion. In total, 80 out of 216 papers made explicit reference to the concept of adjustment. Among them, 30 were published between 2021 and 2016, 12 were published between 2015 and 2011, 14 between 2010 and 2000, and 24 were published before 2000. 

A process of data mapping was carried out to better specify how the literature in refugee studies conceptualizes adjustment. The definition of adjustment was extracted from each of the eligible articles. The emergent definitions were then divided into three categories, according to the specific dimensions of adjustment they addressed. Results from this data mapping process are shown in Table 2. 

The second research question addressed the factors that foster adjustment among adult refugees and their resettlement communities. 

To address RQ2, we conducted a second analysis of the results that emerged from the search protocol adopted to answer RQ1. From the results of the first research question, we were able to broaden the scope of the analysis and include papers that did not explicitly address adjustment, but included other dimensions associated with the conceptualization of adjustment. 

To carry out this second literature analysis, the following inclusion criteria were adopted:

1. Adoption of a positive perspective of adjustment;
2. The reference to an adult population of refugees. This second eligibility criterion arose from the consideration that refugee youth and adults face different challenges related to adjustment. Refugee youth adjustment is mediated by parental adjustment and inclusion in a school context—issues that require a specific level of analysis.

Given the first eligibility criterion, articles that merely focused on adjustment from a pathological perspective were not taken into consideration. Because of this, articles were excluded if they focused on assessment and diagnosis, trauma and psychopathology, mental health, or were conducted in clinical populations. In addition, as we were interested in adult adjustment, articles that focused on youth and intergenerational dynamics were excluded. Lastly, articles that focused on a specific target and goal that differed from the one of the scoping review, and those whose abstracts or texts could not be retrieved, were not considered. 

Twenty articles were then selected to answer RQ2. Among them, 1 was a review, 2 were theoretical papers, and 18 were research studies. In particular: 10 were cross-sectional studies, 2 were longitudinal studies, and 6 were qualitative studies. 

A second data mapping was then carried out in order to answer this research question. Variables associated with the concept of adjustment were extracted from each eligible article. The emergent variables were initially divided into two groups:

1. Those that referred to the resettlement country;
2. Those that referred to refugees.

This second group was then divided into five categories: (a) time—defined as the number of years spent within the resettlement country; (b) social integration markers, according to the Ager and Strang model [18]; (c) acculturation; (d) social support; (e) psychological capital. 

Table 3 shows the results of this second data mapping process.

## 3. Results

### 3.1. RQ1—How Is Adjustment Conceptualized in the Literature?

Within the migration field, studies that explored adjustment referred to the psychological, social, and school dimensions of refugees.

#### 3.1.1. Psychological Dimension

The majority of studies that investigated the psychological adjustment of refugees adopted a pathological paradigm. Indeed, 18 articles considered this kind of adjustment as the mere absence of mental health disorders [26,27,28,29,30,31,32,33,34,35,36,37,38,39,40,41,42,43], in particular, anxiety and depression, while 9 articles specifically addressed the adjustment disorder as a syndrome occurring after an episode of acute stress [44,45,46,47,48,49,50,51,52].

Only seven articles addressed psychological adjustment, according to a positive perspective, defined by the presence of life satisfaction, well-being, perceived health, self-esteem, and optimism [36,53,54,55,56,57,58].

#### 3.1.2. Social Dimension

The social dimension of adjustment explores the social, cultural, and economic inclusion of refugees.

Psychosocial adjustment identifies connections and social support of refugees within the resettlement community. Moreover, 35 articles explored this specific domain of the social dimension of adjustment [28,36,41,53,55,57,59,60,61,62,63,64,65,66,67,68,69,70,71,72,73,74,75,76,77,78,79,80,81,82,83,84,85,86].

The sociocultural adjustment was defined as “the ability of the individual to function in the new culture, in managing daily life tasks and is more related to the social skills acquired in the new society” [87] (p. 66). Sociocultural adjustment therefore refers to the ability of refugees to function within the resettlement community, i.e., being self-sufficient and culturally included. Moreover, 12 articles explored this specific kind of social adjustment [32,58,66,87,88,89,90,91,92,93,94,95].

Lastly, socioeconomic inclusion explores economic inclusion of refugees within the resettlement community, or rather their economic self-sufficiency as a result of job attainment. Only two articles specifically referred to this sub-category of the social dimension of adjustment [43,53]. 

#### 3.1.3. School/Academic Dimension

This dimension of adjustment refers to the outcomes of refugee within a school or in an academic context. Studies addressing school or academic adjustment have been conducted within refugee youth populations. In particular, five studies explored school adjustment [96,97,98,99,100] and four specifically focused on the academic context [57,64,72,92].

All of these studies explored specific, yet interrelated dimensions of adjustment. By doing so, they only offered partial definitions of what adjustment is.

Furthermore, these three dimensions see adjustment as a process at the refugee’s expense. No papers mentioned the dialectic among the psychological, social, and cultural inclusion of refugees, and community development. Thus, the representation of refugees that emerge from these papers is the one of foreigners, who, notwithstanding their ability to include themselves in the local community, do not actively contribute to community development.

### 3.2. RQ2—What Are the Factors That Foster the Adjustment among Refugees and Their Resettlement Communities?

The second research question addresses the factors that eased the adjustment among adult refugees and their resettlement communities, according to a positive perspective. Data mapping identified six categories of factors that foster adjustment. Each macro category will be described into detail by exploring the categories they consist of. Each category represents one factor that fosters refugee adjustment within the local resettlement country.

#### 3.2.1. Context Characteristic

As Berry [20] pointed out, the acculturation strategies of both the resettlement country and newcomers are inter-related. A country that chooses (or not) to foster social, cultural, and economic inclusion within its local communities influences newcomer adjustment. 

According to the results of our scoping review, four papers specifically investigated the resettlement context characteristics that ease the adjustment of refugees. 

##### Acculturation Strategies

Referencing the acculturation theory—Beiser’s experience [66] outlined that the Canadian multiculturalist acculturative strategy, formalized through the Multiculturalism act of 1988, fostered the refugees’ maintenance of the heritage culture and participation in the larger resettlement community. In other words, the author recognized a correspondence in the acculturation strategy adopted by the resettlement country and the one adopted by refugees. 

##### Resettlement Policies

Kahn [101] addressed the relationship between the cultural values of countries, in terms of inclusion and immigration policies. In particular, he studied cases from the U.S. and Germany. According to the U.S. Constitution, as an immigration country, the U.S. fosters a feeling of civic nationalism, characterized by the possibility of affiliation, to have rights granted, despite geographical provenience. Unfortunately, as the author highlights, despite this cultural background, U.S. acceptance policies have become stricter in recent years, so a mismatch emerges between the cultural values and political decisions. On the contrary, Germany is characterized by an ethnic nationalism that, on the one hand guarantees citizenship to German descendants, while on the other hand, makes it more difficult for non-German people to be socially included in the country. Nevertheless, Kahn outlines that German law, different from the U.S., has adopted strategies to increase the number of refugees to be welcomed in the country. 

Even Tress [81] confronted the U.S. and Germany cases with specific regard to the reception and adjustment of Jewish refugees after the fall of the Soviet Union. The author reviewed the details of the labor market and the refugee resettlement policies to verify their effects on refugee adjustment, especially from an employment point of view. According to the author, the American market-oriented resettlement system fostered higher economic self-sufficiency when compared to the effects of the German policy-based resettlement system. The study also highlights that neither country, with their different resettlement systems, allowed Jewish refugees to attain the same pre-flight employment conditions. On the contrary, in both situations, participants emerged as having lower employment conditions. Further results, presented in the following paragraphs, will testify to the positive impacts of job satisfaction in regard to refugee adjustment. 

Beiser [66], consistent with research conducted by Tress [81], also studied Canadian policies for employment of refugees. The author outlined that the refugees who benefited from the Canadian practices of private sponsorship obtained job positions more easily than those sponsored by the government. In addition, privately sponsored refugees received higher support (regarding sociocultural adjustments) and had a wider network of relationships with the autochthonous than those who were sponsored by the government. 

##### Place of Relocation

Al-Srehan [79] explored the Syrian refugee perceptions, regarding the effectiveness of the Jordan resettlement policies. According to the study results, those who resettled in a city had a “higher effectiveness” perception of the resettlement policies compared to those who resettled in a village. This result shows that refugees who resettle in the same country can have different adjustment experiences, depending on where they are relocated (i.e., in a small or in a big center). 

#### 3.2.2. Time Spent in the Resettlement Country

The number of years spent within the resettlement country is cited as a factor influencing the adjustment of refugees in five out of twenty studies [35,53,79,102,103]. Time had a positive impact on employment, financial and residency conditions, access to healthcare services, engagement in social relationships (both with the heritage and the resettlement community), and on life satisfaction. Nevertheless, one study [35] found that the time spent within the resettlement country fostered mental distress. The same study highlighted that the number of years spent within the resettlement country fostered the acknowledgment of the resettlement culture that, in turn, diminished mental distress. 

#### 3.2.3. Refugee’s Social Integration Markers

##### Employment

Three out of twenty studies cited employment as an element that fostered the adjustment of refugees [33,56,91]. 

Interestingly, two studies [33,91] explored the employment conditions, not just considering the occupational status, but also considering whether the occupation was a source of satisfaction. Indeed, as Salo and Birman highlight, “many refugees’ current employment—even when considered high status or well paid by objective measures—may be lower than their pre-migration work status or not in line with their professional identity” [33] (p. 397). Job satisfaction emerged to foster psychological adjustment, here defined by the absence of psychopathology, and an active involvement in the resettlement community. Lastly, research by Wallin and Ahlström’s [56] suggest that occupational status fostered self-reliance and autonomy in refugees, as well as a deeper connection with the resettlement community. 

##### Education

From our scoping review, one study mentioned education as a factor that positively influences adjustment [53]. According to this study, higher educational levels were positively associated with occupational status, the establishment of relationships with resettlement community members, and health. Nevertheless, the study highlighted that the higher the level of education, the lower the refugee’s life satisfaction and self-esteem. Consistent with previous outcomes on the employment conditions, the authors hypothesize that these last results could be explained by considering that the refugees they considered were unable to rebuild their previous occupational and professional conditions within the resettlement community. 

##### Housing

One out of twenty studies explored the housing dimensions [27]. According to the study by Haase and colleagues, refugees living in an apartment had better psychological adjustment than those living in resettlement centers. This relationship was mediated by perceived discrimination: indeed, those living in reception centers reported higher levels of perceived discrimination that in turn affected their psychological adjustment. This study defined psychological adjustment as the absence of psychological problems.

#### 3.2.4. Refugees’ Acculturation

Acculturation was studied, as a whole strategy approach and maintenance of the resettlement and heritage culture, as well as in the components of identity, language, and behavior. 

##### Strategy

Regarding the process of acculturation, El Khoury [87] found that both integration and assimilation strategies [20] foster sociocultural adaptation [87]. Nevertheless, the author also highlighted that, whilst integration, characterized by the engagement in the resettlement and heritage culture, fostered psychological well-being—assimilation, which is instead characterized by the loss of contact with the heritage culture, was associated with low levels of psychological well-being. Two other studies testified that the acknowledgement of the resettlement culture was related to higher levels of psychological adjustment, considered as the absence of psychopathology [33,35] and life satisfaction [35]. In both studies, the relationship between acculturation and psychological adjustment was mediated by occupational status.

With regard to the maintenance of the heritage culture, Salo and Birman’s study [33] showed that this kind of acculturation fostered the co-ethnic social support satisfaction, defined as a perceived satisfaction in the resourcefulness of the co-ethnic networks in providing social and financial support and enjoyment. Nevertheless, this study also found that heritage acculturation was positively associated with job satisfaction. In addition, Birman and colleagues [35] highlighted that the maintenance of the heritage culture also reduced psychological distress through the mediation of the aforementioned co-ethnic social support satisfaction and life satisfaction.

##### Identity

With reference to identity, Kahn’s [101] theoretical paper identified three identity stages that brought cultural adjustment—immersion, biculturalism, and transculturalism. The first stage is characterized by superficial changes that occur when a person enters a new community. The author sustains that, within this stage, people who are eager to abandon their heritage culture may idealize and immerge into the resettlement culture, usually facing a disillusionment. On the contrary, people reluctant to encounter the resettlement culture might instead hold on to the heritage culture. According to Kahn, the immersion stage is frequently followed by biculturalism, whereby a person recognizes signs of belongingness to both the heritage and the resettlement culture. Within the last stage, a person deeply experiences a sense of belongingness to both cultures. When this final stage is achieved, identity acquires complexity and generates creativity, especially in the employment field through entrepreneurship. 

##### Language

The impact of language on the adjustment of refugees was studied in four out of twenty studies [39,53,56,87]. Results show that acquisition of the resettlement community language fosters sociocultural adjustment [87], employment, contact with the resettlement community [53], and psychological adjustment defined both by the absence of psychopathology [39] and by the perception of personal well-being and inner resources [53,56]. Interestingly, no study specifically addresses the effect of the acknowledgement and use of the heritage language on adjustment.

##### Behavior

Behavioral acculturation was studied as the enactment of behaviors aimed at approaching the resettlement culture and maintaining the heritage. 

Birman and Tran [39] explored the factor that fostered the adjustment of a group of Vietnamese refugees who resettled in the U.S. With specific regard to the behavioral dimension of acculturation, the authors found that American behavior predicted a reduced perception of cultural alienation, defined as “a sense of estrangement from the surrounding culture rather than a sense of connection, belonging and at home in their new culture and country” [39] (p. 111). The results concerning Vietnamese behavioral acculturation are contradictory. On the one hand, this dimension predicts life satisfaction, and on the other hand, it predicts anxiety.

##### Sense of Community

Sense of community is usually not considered part of acculturation. Nevertheless, because it is related to dynamics of belongingness and community participation, we consider this dimension as a late and final stage of acculturation toward the resettlement community. McMillan and Chavis [104] defined four dimensions of the sense of community: *membership, influence, integration, and fulfillment of needs, and shared emotional connection* (p. 9). *Membership* refers to a sense of belonging; *influence* refers to a sense of efficacy; *integration and fulfillment of needs* relates to the belief that the resources produced by virtue of membership will address the community needs; lastly, *shared emotional connection* addresses the members’ awareness of sharing common history, values, and experiences. Interestingly, just one theoretical paper [55] addressed the impact of sense of community on refugees’ adjustment. According to previous studies conducted in a non-refugee population, Townley and colleagues [55] suggest that sense of community may increase refugees’ psychological well-being, perception of belonging, community connectedness, and involvement in the community. Further studies should be carried out to confirm the authors’ suggestions.

#### 3.2.5. Refugees’ Social Support

We identified two networks of support that ease refugees’ adjustment within the resettlement community: the heritage community and the resettlement community. 

##### Heritage Community

Six out of twenty studies highlighted the resourcefulness of co-ethnic networks for refugees’ adjustment within the resettlement community. According to Williamson [91], a common destiny, language, and living in the same residential area, is what brings together refugees from the same country. Co-ethnic networks foster sociocultural adjustment by fostering financial, health, employment, and practical support [69,105]. Results show that co-ethnic networks also promote refugees’ psychological adjustment by fostering emotional and decision-making support [105], life satisfaction [39], and reducing loneliness [56]. Results on the impact of these relationships on mental health are instead heterogenous: indeed, they reduce depression, but have no impact on anxiety [39]. Interestingly, results show that refugees’ connections with the heritage community also foster integration from an acculturative point of view; indeed, they promote attachment to the heritage community, ease the connection with the resettlement community [106], and ease the acquisition of the resettlement language [69].

##### Resettlement Community

Results on the impact of connections with the resettlement community are more exiguous. Studies addressing this issue highlight that the connections with the resettlement community members impact refugee perceptions of being welcomed and accepted within the local community. According to Haase and colleagues [27], experiences of positive contact with the resettlement community members foster a higher desire for intergroup contact and the perception of the resettlement country as welcoming. Similarly, in Hansen’s [69] study, connections with the resettlement community provided the feeling of being accepted within the local community in a group of Bosnian refugees living in North Dakota. Lastly, Smith [105] found that relationships with the resettlement community also fostered practical support for resettlement. 

#### 3.2.6. Refugees’ Psychological Capital

Results on the impact of psychological capital, defined by Youssef-Morgan and Luthans as “an individual’s positive psychological state of development” [107] (p. 181), are more fragmented and difficult to be charted. This preliminary result suggests that this area should be the object of further investigations.

Hahn and colleagues [53] investigated the role of personal characteristics, such as internal locus of control, willingness to reciprocity, and a risk-taking attitude in easing refugees’ adjustment. According to their results, a risk-taking attitude and an internal locus of control fostered a wider social network. In addition, an internal locus of control and an attitude toward reciprocity promote a psychological adjustment, defined by the presence of life satisfaction and self-esteem. Lastly, the risk-taking attitude is positively correlated to stable employment conditions. 

In Henry’s qualitative study [93], results show that, among a group of refugees from Sub-Saharan Africa (Burundi, Somalia, Ethiopia, Sudan), those who reported “optimism, self-reliance, compassion, rejection of the victim role and a positive attitude toward education and openness” (p. 599) could elaborate the physical detachment from the heritage culture and develop a new, yet continuative bond with it. This process allowed them to confidentially approach the resettlement culture and ultimately integrate the two. Within this framework, the inner resources fostered an acculturative strategy characterized by integration.

In Hansen’s qualitative study [69], consistent with the aforementioned studies, it emerged that proactivity was an element that fostered the adjustment of a group of Bosnian refugees who resettled in North Dakota (USA). 

Lastly, Stoll and Johnson’s [67] cross-sectional study highlighted that religiosity, defined as “relatedness to an ultimate being” (p. 629), fostered the psychological adjustment of a group of Sudanese refugees who resettled in Canada.

## 4. Discussion

This scoping review—against a branch of studies that considers migration as a source of pathological conditions—was aimed at tracking the state-of-the-art literature on the issue of refugees’ adjustment within their resettlement communities.

We considered adjustment as a complex and continuative process that evolves according to refugees’ progressive participation to the local communities. Nevertheless, studies in the field generally consider adjustment, solely referred to as the process of refugee adaptation within the resettlement community. The psychological adjustment is indeed addressed as the impact of the adaptation process on refugees’ mental health and well-being. The social adjustment focuses on refugees’ commitments toward social, cultural, and economic inclusion. Lastly, school adjustment explores young refugees’ performances in schools. Even though the literature recognizes that adjustment is the result of a dialectic among the resettlement country’s cultural, political, and economic features, and a refugee’s efforts to become socially and culturally integrated, results of this relationship are investigated merely with regard to the adaptation of refugees. The impact of adjustment on the local community’s development is not explored; thus, confirming the difficulty in considering refugees as a resource for local territories. 

Another consideration that needs to be conducted involves the fragmentation of the concept of adjustment. As above-mentioned, the literature addresses refugees’ adjustment from three different vertexes—psychological, social, and scholastic. Nevertheless, when analyzing the factors that foster adjustment among refugees and the local communities, results show that these different dimensions are instead strongly inter-related. We therefore propose that, in order to be fully understood, they should be investigated as parts of the same phenomenon. 

Regarding the adjustment perspectives of refugees, the majority of studies that address psychological adjustment adopt pathological perspectives. Indeed, eighteen articles defined this dimension of adjustment as the mere absence of mental health disorders while nine more studies specifically addressed the adjustment disorder. On the contrary, just seven studies defined psychological adjustment in a resourceful conception as characterized by high levels of quality of life, health, and self-esteem. This consideration demonstrates how difficult it is to study migration through a non-pathological paradigm [108,109].

Adjustment varies according to the community whereby it takes place. As Berry sustained in 1997 [20], the receptive culture of the resettlement country influences the way refugees adjust. Therefore, when studying such a phenomenon, it is fundamental not just to consider refugee efforts, but also the contextual characteristics that ease their involvement (or, on the contrary, the obstacles to their involvement) within the resettlement community. 

With this regard, results from our scoping review attest that disconnections may exist among the cultural values of reception and the policies actualized in the same country. While a country may be characterized by the presence of values of cultural inclusion, its political trend may instead follow an opposite direction that restricts the reception of refugees. However, countries, such as Germany, characterized by ethnic nationalism, are instead committed toward a wide reception of people fleeing their homes because of violence. Good receptive practices have also emerged; examples from Canada show that refugees’ private sponsorships ease job attainment, the connection with the resettlement community, and the sociocultural adjustment, in general.

Results show that, not only do the reception politics influence refugee adjustment, but also those related to the labor market. Employment became particularly critical in terms of adjustment. The majority of studies that investigated this specific aspect outlined that refugees could hardly attain the same “pre-flight” job positions; nevertheless, those who were satisfied with their job positions saw an increase in terms of both sociocultural and psychological adjustment. The impossibility to achieve the same pre-flight job position pushes refugees toward entrepreneurship, an issue that connects the employment dimension with the psychological–capital dimension.

Organizational psychology has outlined that refugees, given their migration experience, are characterized by high levels of self-efficacy and risk-taking attitudes, resources that they frequently pour into entrepreneurship experiences [109,110]. This scoping review identified the area of psychological capital as a field that needs further investigation. The fact that this dimension is still lacking confirms again the pathological approach that has characterized, until now, research in the migration area [11] and the social representation of refugees as resourceless people.

Another aspect that clearly emerged from the literature review is the one related to the time spent within the resettlement community. Time is associated with better socio-cultural and psychological adjustments. Such a result confirms the hypothesis that adjustment is a continuative process that, in order to be effectively studied, requires a long-term perspective. Connected to this issue is the dimension of housing. Although the effect of housing conditions on adjustment is poorly investigated, results testify that once out of the resettlement system and relocated in a house-for-rent, refugees who resettled in Germany perceived lower discrimination and, consequently, reported improved mental health conditions.

The neighborhood of the resettlement is also important as it is strongly relates to the kind of social support refugees receive. Living in a neighborhood inhabited by members of the heritage community allows refugee to access what, in sociology, has been addressed as bonding social capital, or rather resourceful connections, in terms of trust and cooperation among people who feel that they belong to the same social group; in this case, the heritage community [111]. Consistent with the literature on bonding social capital in the migration area [6,18,112,113,114,115], results from the scoping review show that the connections with the heritage community provide refugees with emotional, financial, and practical support. Participation in the heritage community also fosters employment opportunities and sociocultural and psychological adjustment. Interestingly, our results outline that the heritage community can function as a bridge for the constitutions of relationships with the resettlement community. Relationships with the resettlement community in turn are fundamental toward developing a sense of belonging in this community. Sociological literature in the migration field addresses the resourceful relationships among newcomers and the resettlement community as bridging social capital [116]. Interestingly, sociology considers bridging social capital as the stock of resources produced through connections among people who feel that they belong to a different social group [111]. Our scoping review highlights that bridging social capital is exactly the resource that fosters a common belongingness among newcomers and the autochthonous.

This last issue involves the dimension of acculturation. Results show that integration strategies [20] foster both sociocultural and psychological adjustment. In addition, studies attest that the acculturation dimension is strongly inter-related with the other factors leading refugee adjustment, in particular with employment and social support. Such a result testifies that the sociological and psychological dimensions of adjustment are inter-related, and that, in order to be fully understood, need to be studied in their interaction. Literature that specifically addresses the identity dimension of acculturation shows the positive impact of integration strategies on the mental health and social integration of refugees [117]; interestingly, Kahn’s psychoanalytical theoretical paper [101] addressed the deployment of an identity process that brings such a result. The author outlines three stages that end with a deep-felt sense of belonging to both the resettlement and the heritage community. Khan also highlighted that such a stage is a source of creativity in the employment field, especially through entrepreneurship, an issue that connects the acculturation dimension with sociological integration and psychological capital. 

Within acculturation, language is the aspect most explored. The ability to speak both the heritage and the resettlement language is what connects refugees to both their communities of belonging, bonding and bridging social capital. 

Consistent with a social representation that sees refugees as perennial foreigners within their resettlement communities, no studies were found to explore their active engagement within the local communities and how such an engagement impacts over the adjustment. Only Townley and colleagues [55] address this issue by exploring the construct of the sense of community. The authors hypothesize that sense of community may increase refugees’ well-being, connectedness, and involvement in the community, but studies need to be carried out in order to verify this hypothesis. Sharing a sense of community implies the auto- and hetero-recognition of belongingness to the resettlement community and an active engagement towards its development. Being an active community member implies not just contributing to the community development from an economic point of view, through employment and tax payment, but also from a social and cultural point of view. Studies carried out among populations at risk of isolation showed the benefits of a similar engagement [118,119]. In previous studies, we explored the context of social enterprises with migrant backgrounds [108,109,120], and found that socially integrated new citizens own invaluable human capital, related to the promotion of coexistence among different cultures and social groups. Indeed, new citizens went through an adjustment path, whereby they needed to adapt at least two different national cultures. This experience provided them with an experiential knowledge to foster multiculturalism within their new communities. Because of this, new citizens may play a specific role in the resettlement community’s social and cultural development, placing them in a mutual relationship with the community [121]. Such recognition offers divergent social representation of refugees, in opposition to the one that sees them as foreigners, victims, criminals, and resourceless people. We hypothesize that this process will lead to an improved adjustment for both refugees and resettlement communities. Further studies should verify this hypothesis. 

## 5. Conclusions

To conclude, the present scoping review verified that the literature considers adjustment as a process that impacts the lives of refugees. Even though the context plays a fundamental role on refugees’ social and cultural inclusion, the results of adjustment are not explored from a community perspective. The review also identifies the factors that lead to refugee adjustments within resettlement communities. Importantly, the review shows that adjustment is a complex and continuative process that consists of contextual possibilities and requirements, as well as refugee efforts that require integrate and long-term perspectives in order to be effectively studied. 

According to the results of the scoping review, employment is a central issue for the adjustment of refugees. From a sociological point of view, employment guarantees a self-sufficient position within the community, connecting new and old members of the community. By fostering social connections with the autochthonous, employment also eases the refugees’ approaches toward resettlement culture; therefore, promoting integrative acculturation strategies that allow them to feel/be recognized as effective members of the resettlement community. Lastly, studies demonstrate that a satisfying job increases the self-reliance of refugees and, therefore, self-efficacy, described by Bandura as “people’s beliefs about their capabilities to provide designated levels of performance that exercise influence over events that affect their lives” [122]. This consideration outlines how fundamental it is for resettlement countries to improve labor policies in order to develop inclusive practices for the resettlement of refugees. Best practices should address refugee employment, not just by providing the first job available; on the contrary, they should take into consideration the educational level, past experiences, and attitudes of the referees, to plan tailored employment paths. 

Studies addressing the employment conditions of refugees implicitly recognize their economic contributions to the community. On the contrary, the active contributions of refugees toward the social and cultural development of local communities is a research area that needs further investigation, along with studies on the psychological capital of refugees. By addressing the issues that consider the resourcefulness of refugee experiences, the academia may contribute to a cultural change toward multiculturalism and inclusiveness.

## Figures and Tables

**Table 1:** Inclusion and exclusion criteria of the search protocol.

Criterion	Inclusion	Exclusion
Years	No time limit	-
Language	English	Non-English articles
Type of articles	Scientific papers: quantitative, qualitative, mixed method, review, theoretical	Non-scientific papers

**Table 2:** Adjustment dimensions.

Dimension	Sub-Category
Psychological	Absence of mental disorders
	Adjustment disorder
	Positive definition
Social	Psychosocial
	Sociocultural
	Socioeconomic
School/academic	-

**Table 3:** Factors that foster adult refugee adjustment within resettlement communities, data mapping.

Macro Category	Category
Context characteristics	Acculturation strategies
	Resettlement policies
	Place of relocation
Time spent in the resettlement country	-
Refugee’s social integration markers	Employment
	Education
	Housing
Refugee’s acculturation	Strategy
	Identity
	Language
	Behavior
	Sense of Community
Refugee’s social support	Heritage community
	Resettlement community
Refugee’s psychological capital	-
